# Learning fast accurate movements requires intact frontostriatal circuits

**DOI:** 10.3389/fnhum.2013.00752

**Published:** 2013-11-13

**Authors:** Britne Shabbott, Roshni Ravindran, Joseph W. Schumacher, Paula B. Wasserman, Karen S. Marder, Pietro Mazzoni

**Affiliations:** ^1^Motor Performance Lab, Department of Neurology, The Neurological Institute, Columbia UniversityNew York, NY, USA; ^2^Huntington's Disease Center of Excellence, Columbia University Medical CenterNew York, NY, USA; ^3^G. H. Sergievsky Center, Columbia University Medical CenterNew York, NY, USA; ^4^Departments of Neurology and Psychiatry, Columbia University Medical CenterNew York, NY, USA

**Keywords:** motor skill, kinematics, reaching, striatum, basal ganglia, movement disorder, neurodegenerative, neurological

## Abstract

The basal ganglia are known to play a crucial role in movement execution, but their importance for motor skill learning remains unclear. Obstacles to our understanding include the lack of a universally accepted definition of motor skill learning (definition confound), and difficulties in distinguishing learning deficits from execution impairments (performance confound). We studied how healthy subjects and subjects with a basal ganglia disorder learn fast accurate reaching movements. We addressed the definition and performance confounds by: (1) focusing on an operationally defined core element of motor skill learning (speed-accuracy learning), and (2) using normal variation in initial performance to separate movement execution impairment from motor learning abnormalities. We measured motor skill learning as performance improvement in a reaching task with a speed-accuracy trade-off. We compared the performance of subjects with Huntington's disease (HD), a neurodegenerative basal ganglia disorder, to that of premanifest carriers of the HD mutation and of control subjects. The initial movements of HD subjects were less skilled (slower and/or less accurate) than those of control subjects. To factor out these differences in initial execution, we modeled the relationship between learning and baseline performance in control subjects. Subjects with HD exhibited a clear learning impairment that was not explained by differences in initial performance. These results support a role for the basal ganglia in both movement execution and motor skill learning.

## Introduction

While the basal ganglia have long been implicated in motor learning (Knowlton et al., 1996; Yin and Knowlton, 2006), their role in this type of learning remains incompletely understood. The basal ganglia are active during acquisition and retrieval of motor habits in rats (Jog et al., 1999; Howe et al., 2011), and during acquisition and recall of motor sequences (Orban et al., 2010) and novel actions (Monchi et al., 2006) in humans. Pharmacological disruption of striatal activity prevents normal performance of learned sequences (Miyachi et al., 1997; Eckart et al., 2010). On the other hand, a large number of studies on motor learning in patients with either of two basal ganglia disorders [Parkinson's disease (PD) and Huntington's disease (HD)] have yielded mixed results. These studies, reviewed in the Discussion, include demonstrations of both disrupted and intact motor learning in patients with PD and patients with HD.

Some of the difficulty in interpreting previous studies stems from the fact that the term “motor learning” has been used to refer to different types of learning, including motor adaptation, sequence learning, and motor skill learning (Krakauer and Mazzoni, 2011). These types of learning represent distinct computational processes that may engage different neural substrates. In addition, neurologic disorders can disrupt basic movement execution. This disruption poses a challenge in studying motor learning in patient populations because abnormal movement execution could affect motor learning (Soliveri et al., 1997). The basal ganglia are important for normal execution of well-rehearsed movements (Marsden, 1982), and normal learning may thus be masked or impeded by impaired execution.

In this study we examined the role of the basal ganglia in motor skill learning. We focused on a specific component of motor skill: the ability to move fast and accurately. The speed-accuracy trade-off imposes a limit on performance in many daily tasks, and the ability to move faster and more accurately confers an advantage, ecologically as well as in many sports. Indeed, concurrent improvement of speed and accuracy forms the core of several definitions of motor skill learning (Guthrie, 1952; Welford, 1968; Willingham, 1998; Schmidt and Lee, 2005), which can be distilled into “improvement in the quality of motor performance, involving accuracy, speed, and a minimum of energy expenditure” (Sanes et al., 1990).

Motor skill learning is generally thought to include other elements besides speed-accuracy learning, including learning what movements to perform, improving perceptual discrimination, and devising optimal strategies. While the basal ganglia may contribute to more than one of these components (Willingham, 1998; Newell and Vaillancourt, 2001; Liu et al., 2006), here we specifically focused on speed-accuracy improvement. Even though motor skill learning may involve additional processes, these components are potentially subserved by distinct neural processes, including distinct circuits within the basal ganglia, and thus need to be studied in isolation. Speed-accuracy performance reflects the ability to produce movement trajectories with precision and reliability, and thus forms a central element of motor control.

Huntington's disease (HD) is a disorder that causes degeneration of the striatum and related corticostriatal networks (Vonsattel et al., 1985; Rosas et al., 2002; Ross and Tabrizi, 2011). It can serve as a model of disrupted frontostriatal circuits, specifically striatum and cortical motor areas that receive basal ganglia input (Alexander, 1994). We hypothesized that damage to cortico-basal ganglia circuits would affect speed-accuracy learning, as a component of motor skill learning, because such circuits are well poised to modify relationships among movement variables (Albin et al., 1989), and speed-accuracy learning amounts to modifying the trade-off between two competing movement variables. Previous studies that found motor learning impairments in patients with basal ganglia disorders (see Discussion for specific studies) employed tasks that, in our interpretation, tested speed-accuracy learning. We designed a motor learning task that introduced a conflict between speed and accuracy and that did not require motor adaptation or sequence learning. We then applied a novel analytic approach to distinguish motor learning abnormalities from deficits in movement execution.

## Methods

### Subjects

We tested 49 subjects, divided into three groups: 12 subjects with clinically manifest HD (*HD group*); 18 subjects with the HD gene expansion who did not meet criteria for clinical diagnosis of HD (premanifest HD; *prHD group*); and 19 age-matched control subjects (*CTL group*). HD and prHD subjects were recruited through the Huntington's Disease Center of Excellence at Columbia University and included subjects who also participated in the PREDICT-HD study (Paulsen et al., 2006; Biglan et al., 2009). A neurologist administered the motor component of the Unified Huntington's Disease Rating Scale (UHDRS) (Huntington Study Group, 1996) on the day of testing. Inclusion criteria were: for the HD group, a score of 4 on UHDRS question 17 (i.e., 100% confidence in a diagnosis of HD); for prHD, a score ≤ 3 on UHDRS question 17 (Biglan et al., 2009). Control subjects were individuals, recruited from the local community, who had tested negative for the HD gene or had no known family history of HD, and had no known history of neurological or musculoskeletal disease.

We tested prHD subjects, besides HD subjects, because they provided an opportunity to assess whether subtle deficits in motor skill learning emerge prior to the time of clinical diagnosis. Such findings may enhance our understanding of the mechanisms of symptom onset in HD. In addition, prHD subjects might demonstrate a dissociation between initial performance impairments and learning deficits, which could inform the extent to which these disruptions might be independent.

Subject characteristics are listed in Table 1. There was no significant difference in age among the three subject groups (*p* = 0.45; ANOVA). All participants were free of cognitive impairment (Mini-mental Status Examination score >27; Folstein et al., 1975). Three HD subjects and two prHD subjects were taking antidepressant medications (selective serotonin reuptake inhibitors), but no subject was taking medications that might impair motor function, such as neuroleptics or benzodiazepines.

**Table 1:** **Subject Characteristics**.

**Group**	**Age**	**Gender**	**UHDRS**	Chorea^*^
CTL	20	F	NA	NA
CTL	25	F	NA	NA
CTL	27	F	NA	NA
CTL	28	F	NA	NA
CTL	29	F	NA	NA
CTL	33	M	NA	NA
CTL	33	F	NA	NA
CTL	36	M	NA	NA
CTL	37	F	NA	NA
CTL	38	M	NA	NA
CTL	38	F	NA	NA
CTL	39	M	NA	NA
CTL	43	F	NA	NA
CTL	44	F	NA	NA
CTL	44	F	NA	NA
CTL	46	F	NA	NA
CTL	51	F	NA	NA
CTL	56	F	NA	NA
CTL	58	M	NA	NA
prHD	30	F	0	0
prHD	36	F	1	0
prHD	36	M	2	1
prHD	47	F	2	0
prHD	64	F	2	0
prHD	26	M	3	0
prHD	32	F	3	0
prHD	36	F	3	0
prHD	27	F	4	0
prHD	45	F	4	0
prHD	54	M	4	0
prHD	43	M	5	0
prHD	45	F	5	0
prHD	39	M	6	0
prHD	34	M	8	0
prHD	35	M	8	0
prHD	45	M	8	0
prHD	66	F	8	0
HD	58	M	10	0
HD	65	M	14	0
HD	47	M	15	1
HD	30	M	19	2
HD	35	M	20	2
HD	44	M	23	2
HD	61	F	29	2
HD	32	M	32	2
HD	33	M	36	2
HD	40	M	39	1
HD	33	M	44	0
HD	41	M	67	2

* Score for “Maximal chorea” item for the dominant upper extremity (i.e., the hand and arm tested in the present study) in the UHDRS. 0, absent; 1, slight/intermittent; 2, mild/common or moderate/intermittent; 3, moderate/common; 4, marked/prolonged.

All subjects signed an informed consent, which was approved by Columbia University's Institutional Review Board. Testing was performed in accordance with the Declaration of Helsinki.

### Apparatus

**Figure 1:**
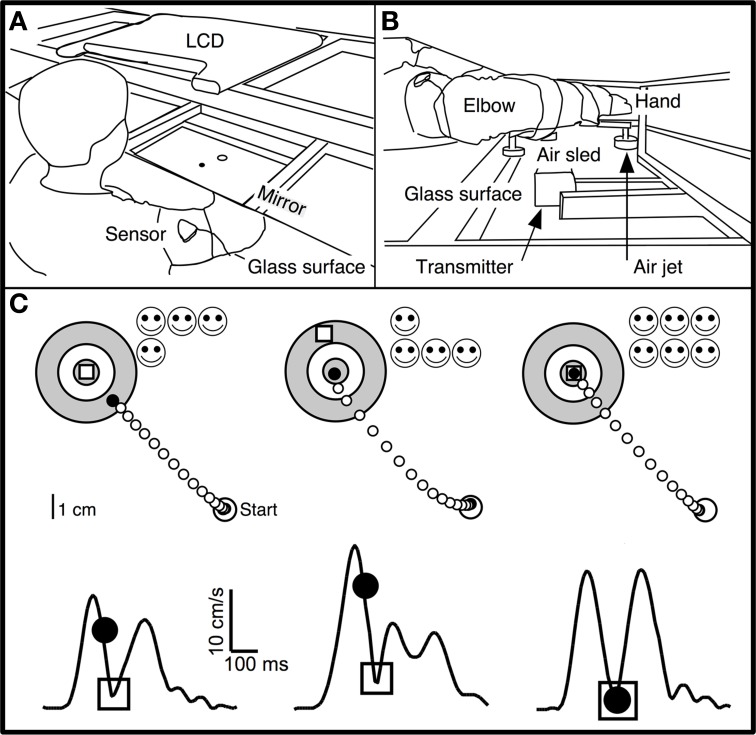
**Experimental apparatus and skill task. (A,B)** Subjects were seated at a table with their dominant arm strapped to a support (air sled) that was lifted slightly off the table by compressed air jets, which allowed frictionless planar arm motion. They viewed the display of an LCD screen, reflected by a mirror located above their arm. The middle finger's tip position was displayed as a cursor (black circle) whose virtual image in the mirror coincided with the fingertip's physical location. Magnetic sensors on the upper arm and forearm recorded position of fingertip, elbow, and shoulder. **(C)** Spatial (upper panels) and temporal (lower panels) illustration of the double-marker task. The subject's hand moved from the start circle toward the target. The handpath (white circles) was not visible to the subject. After 200 ms, a “timed disc” (black circle) appeared at the middle fingertip's location at that time. An “endpoint square” (white square) appeared at the location where the hand reversed direction and returned toward the start circle. Subjects were instructed to place both markers into the center of the target. Faster movements caused the timed disc to be placed closer to the target. This method translated speed into spatial performance with the same metrics as endpoint accuracy. The timed disc's distance from the target indicated a “speed error,” while the endpoint square's distance indicated endpoint error. The sum of these errors was a measure of “skill error.” The tangential velocity profiles (hand velocity vs. time) in the lower panels show the timing of these events during single movements, with the timed disc appearing after 200 ms and the endpoint square appearing at the time of the first velocity minimum. *Left*: Sample trial with small endpoint error and large speed error. *Middle*: Large spatial error and small speed error. *Right*: Small spatial and speed errors; timed disc and endpoint square both landed within the target center.

The apparatus was designed for kinematic studies of planar, reaching-like arm movements to visual targets, as previously described (Mazzoni et al., 2007). Subjects sat at a glass table and made frictionless movements with their dominant arm strapped to an air-jet sled (Figures 1A,B). Fingertip position was collected at 120 Hz using a magnetic motion-tracking system (Flock-of-birds, Ascension Technology), and was displayed on a computer monitor. Subjects viewed this monitor's reflection in a horizontal mirror suspended over the workspace at a height that made the monitor's image appear in the plane of the hand. Subjects thus monitored their hand's position by looking in the mirror at a screen cursor whose image coincided with the position of their fingertip (veridical display).

### Double-marker skill task

Subjects made out-and-back planar reaching movements from a central starting location (“start” circle) to one of 16 targets (circles of 0.5 cm radius, surrounded by two concentric rings in a “bull's eye” pattern; Figure 1C) in a circular target array. The distance from start location to targets was 8 cm.

To start a trial, subjects moved their fingertip into the start circle at the center of the target array, being aided by a cursor (a filled circle of 0.2 cm radius) that indicated fingertip position and that appeared when the fingertip was within a 3 cm distance from the center of the start circle. After the cursor spent 500 ms in the start circle, one of the 16 targets changed color from gray to green and a gentle tone was played. Subjects were instructed to make an out-and-back reaching-like movement to the target. The were told to start the movement when they felt ready (not as soon as possible). Therefore, the task was not a reaction-time task.

A trial began when the fingertip exited the start circle, at which time the cursor disappeared. The movement was thus made without ongoing visual feedback. Visual feedback was provided as two “markers”: a white square and a black disc (Figure 1C). The white square (*endpoint square*) appeared as a static white square when the hand reversed direction (i.e., when radial velocity reversed changed from positive to negative) and indicated finger position at the end of the movement. The black disc (*timed disc*) appeared as a static filled black disc 200 ms after the fingertip exited the start circle. It showed the hand's position after a fixed time interval and represented a position cue indicating movement speed. The trial ended 500 ms after both markers appeared (typically, the timed disc appeared first), at which time the two markers disappeared and a 1 s delay was imposed, after which subjects were allowed to start the next trial.

The goal (and the instruction to the subject) was to place both markers into the center of the target. The two markers imposed accuracy and speed requirements: the endpoint square indicated the accuracy of the movement's endpoint, and the timed disc indicated whether the movement had been made at the correct average speed (Figure 1C). Smiley faces were shown after each trial to indicate marker accuracy (Figure 1C): 3 faces for a marker in the center circle (distance from target center <0.5 cm); 2 faces for the middle ring (distance >0.5 and <2.25 cm); 1 face for the outer ring (distance >2.25 and <4 cm). Thus, up to 6 smiley faces (3 for each marker) could be obtained for each trial. Subjects were not explicitly informed regarding which row of smiley faces corresponded to each marker.

The *double-marker skill task* imposed a speed-accuracy trade-off by requiring subjects to make movements that were both fast (to place the timed disc into the target) and accurate (to place the endpoint square into the target) (Figure 1C, right). In most previous speed-accuracy trade-off tasks (Plamondon and Alimi, 1997), the trade-off between speed and accuracy was not experimentally defined, and speed and accuracy led to different components of task success. We considered it important for subjects to have immediate and clear feedback on the relative contribution of speed and accuracy to task success. The double-marker task achieved this goal by converting speed performance into a spatial error (distance between timed disc and target). Speed performance thus had the same metric as spatial accuracy, and the trade-off between speed and accuracy was obvious as the relative distance of the two markers from the target.

### Learning session

After 24 familiarization trials, subjects made movements to each of the 16 targets in pseudorandom order during the learning session, which consisted of 4 blocks of 64 trials each (256 trials total), with 15 s breaks between blocks.

### Kinematic analysis

Hand position data was low-pass filtered at 8 Hz and differentiated to yield tangential velocity and acceleration. Peak velocity was identified as the first velocity peak after it crossed a 10 cm/s threshold. Movement onset was the last time that velocity crossed a 2 cm/s threshold prior to peak velocity. Movement end was the time, after the time of peak velocity, when radial hand velocity changed sign from positive to negative (i.e., when the hand reversed direction from outgoing to returning toward the start circle). *Endpoint error* was the distance between endpoint square and target. S*peed error* was the distance between timed disc and target. We defined s*kill error* as the sum of endpoint error and speed error. This definition matched the task's reward structure, because the number of smiley faces shown was proportional to how close each marker was to the target. This quantity provided a measure, more finely graded than the number of smiley faces, of how closely subjects achieved the task goal, which could result from various combinations of speed and accuracy.

For selected calculations, data was grouped into 8 *epochs* of 32 trials each. *Delta skill error* was the difference in average skill error between the first and last epochs. *Delta percent skill error* was the delta skill error divided by the average skill error for the first epoch. For selected figures and calculations (as noted below), endpoint error, speed error, and skill error were smoothed with a 16-trial moving average (boxcar smoothing, with end effects handled via the “bounce” approach). Analysis was performed with custom routines in Igor Pro (Wavemetrics).

### Global regression models

We developed a modeling approach to remove the confound of differences in initial performance between subject groups. We expected, based on previous studies, that HD subjects might differ in their initial performance from the CTL group. When learning is compared between groups with different initial performance, it is not clear *a priori* how initial-performance differences should be handled. We examined the above-defined measures of delta skill error and delta percent skill error, i.e., both raw and fractional changes, as measures of learning. However, we wanted to move beyond an arbitrary choice of one of these methods. We started by explicitly considering the assumptions that underlie selection of raw vs. fractional performance changes. We then employed a model-based analysis that exploited individual variation to test the validity of these assumptions. Raw changes are appropriate if we assume that learning is *independent* of initial performance, that is, performance improves at the same rate regardless of its initial value. The relationship between error and trial number is in this case linear. Fractional changes are appropriate if performance changes are *proportional* to initial performance, that is, the relationship between error and trial number is a decaying exponential (a property of the exponential function is that it has a linear relationship with its slope).

The models assumed an underlying learning process that characterized each group as a whole, while permitting individual subjects to start at a unique position along that process, based on their initial performance. Such models were not designed to capture other inter-subject variation, such as individual learning rates or asymptotic performance. Our goal was only to extract group-level learning performance while removing the confounding effect of inter-individual differences in initial task performance. We thus hypothesized an underlying global relationship (linear or exponential) with group-level variables for the learning process, while allowing subject-specific values for initial performance. We used global regression to determine the underlying learning rates (slope for linear model; time constant for exponential model) and performance after extensive practice (asymptote for exponential model) as group variables, while allowing for subject-specific variables for initial performance (y-intercept for linear model; amplitude for exponential model). A global model defines a family of curves, rather than just a single curve. Some parameters are shared so a single parameter value applies to all the curves, while other parameters apply to each data individually.

We performed global fit analyses for each group separately, using the time course of smoothed skill error data. We used smoothed data to reduce the contribution of short-term variations to model convergence. Our interest was in the overall learning process and not in trial-by-trial fluctuations. We tested these skill learning models using the global curve fitting function in STATA, version 10 (StataCorp). We modeled skill error both linearly,

(1) $$\text{Skill }{\text{Error}}_{\text{lin}.}=r\cdot T+b$$

and exponentially,

(2) $$\text{Skill }{\text{Error}}_{\text{exp}.}=\text{C}+\alpha \cdot e^{-T/\tau }$$

where *T* represents trial number, and τ is a time constant. For the linear global model, we set initial error (*b*) as subject-specific variables, while the learning rate (*r*) was a single group-level variable. The number of free parameters was thus N + 1, where N is the number of subjects. For the exponential global model, we set the amplitude (α) as subject-specific, while the learning rate (τ) and asymptote (C) were group-wide variables. There were thus N + 2 free parameters in the exponential model. These choices allowed the models to extract each group's time course of learning while allowing for variations in subjects' individual initial performance (*b* for the linear model; α + C for the exponential model). That is, each subject's learning curve was allowed to start at a different level. Model parameters were identified through least-squares minimization of Root Mean Squared Error (RMSE). Goodness of fit was measured with the Akaike Information Criterion, or AIC (Akaike, 1974), a measure that ranks competing models (lower AIC value indicates a better model) while accounting for differences in number of degrees of freedom.

With regards to the exponential model, we chose to allow subject-specific variation in initial performance (amplitude, α) but not in asymptotic performance (asymptote C). We made this choice because all control subjects reached asymptote by the middle of the training session, and the inter-subject variation for asymptotic skill level was negligible. The training session therefore allowed ample opportunity to reach the same asymptote through normal learning, regardless of initial performance.

From a theoretical perspective it would also be difficult to justify setting asymptotic performance as a subject-specific variable, because asymptotic performance is likely affected by variations in the learning process. Here we were interested in discounting inter-individual variations that were not a reflection of learning. Variation, among control subjects, in initial performance likely reflects factors separate from learning ability. For example, different subjects may have had different amounts of practice in tasks that are similar to the task studied here, and may therefore come to the lab with different levels of proficiency. We were not interested in this type of variation in this study, but rather in subjects' ability to follow a normal learning process regardless of their starting skill level. Allowing individual variation in the exponential model's amplitude was a way to discount this type of variation.

In the case of HD subjects, we wanted to discount the effect of the disease on initial performance, in order to better isolate abnormalities in the learning process itself. On the other hand, if HD patients did not reach the same asymptote as control subjects, it would be impossible to distinguish whether the reason was a learning impairment or simply having started with worse initial performance. We take the fact that, as mentioned above, control subjects reached a uniform asymptote well before the end of the learning session, to indicate that, for our task, the asymptote was not appreciably affected by initial performance. The asymptote could thus be taken as a reflection of learning. By setting it as a group-level variable, we introduced the opportunity to detect group-level learning abnormalities through an effect on learning rate, asymptote, or both.

### Analysis of speed-accuracy changes across trials

The correlation between changes in speed and accuracy across trials was calculated by first computing changes in endpoint error and changes in speed error from one trial to the next:

(3) $$\Delta \left(\text{Endpoint Err}.\right)={\left(\text{Endpoint Err}.\right)}_{n\;+\;1}-{\left(\text{Endpoint Err}.\right)}_{n}$$

(4) $$\Delta \left(\text{Speed Err}.\right)={\left(\text{Speed Err}.\right)}_{n\;+\;1}-{\left(\text{Speed Err}.\right)}_{n}$$

where Err. = Error and *n* = trial number. The slope of the linear regression between Δ*(Endpoint Error)* and Δ*(Speed Error)* quantifies the coupling (positive or negative) between changes in accuracy and changes in speed. We refer to this slope as the *speed-accuracy-change slope, S*_Δ*SA*_. We performed this analysis on both raw and smoothed error data (16-trial boxcar smoothing). Both analyses yielded the same patterns of statistical significance. We report results for the smoothed data.

The progression of the correlation between speed and accuracy changes over the course of the learning session was examined by calculating the correlation between Δ*(Endpoint Error)* and Δ*(Speed Error)* at each trial for a 64-trial window centered at each trial. This approach yielded values of speed-accuracy-change slope (*S*_Δ*SA*_) and coefficient of determination (*R*^2^) for trials 32-221 of the 253-trial learning session.

### Statistical analysis

Statistical analysis was performed using JMP, version 6.03 (SAS), and Stata, version 10 (Stata Corporation). The comparisons of interests were chosen *a priori* as HD vs. CTL, and prHD vs. CTL, and were thus performed using *t*-tests (two-sided; significance level α = 0.05). Linear correlation was calculated between motor performance and clinical severity (UHDRS motor score) for HD subjects, and between motor performance and 5-year probability of diagnosis (i.e., 95% probability of meeting criteria for clinical diagnosis within 5 years) (Langbehn et al., 2004) for prHD subjects.

## Results

We will first describe results for CTL and HD groups, and then results for the prHD group.

### General performance

**Figure 2:**
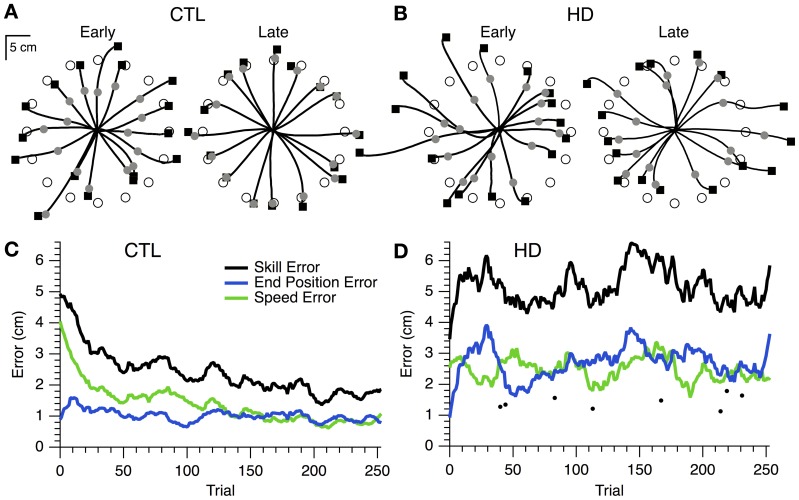
**Hand paths and time course of skill learning for representative single subjects. (A)** Hand paths for a sample CTL subject with the positions of the timed discs (gray circles) and endpoint squares (black squares) shown. “Early” trials (i.e., the first 16 trials of training), left. “Late” trials (i.e., the last 16 trials of training), right. **(B)** Same plots as **(A)**, but for a sample HD subject. **(C)** Smoothed skill error (black), endpoint error (blue), and speed error (green) vs. trial number for a sample CTL subject. **(D)** Same plot as **(C)**, but for a sample HD subject. Single trial examples of skill error (filled circles) are also shown.

The initial performance of single CTL and HD subjects is shown in the hand paths for single movements to 16 targets (Figures 2A,B, left). The timed discs fell short of the target, indicating that movements were initially too slow. Final position was also inaccurate, as shown by the endpoint squares overshooting some targets and undershooting others. For the CTL subject, both speed and position errors became smaller after the learning session (Figure 2A, right). The time course of errors (Figure 2C) revealed a steady decrease in speed and position errors, and in their sum (skill error) throughout the practice session.

In contrast, the hand paths of the single HD subject showed little improvement from first to last cycle (Figure 2B). The time course of skill error for this subject remained relatively stable throughout the learning session (Figure 2D), and was overall greater than skill error for the sample CTL subject (Figure 2C). Interestingly, each component of skill error showed periods of improvement during the course of practice coincident with a decline in performance for the other component of skill error (Figure 2D). The difficulty was thus not in reducing position or speed error individually, but rather in reducing the trade-off between speed and accuracy.

**Figure 3:**
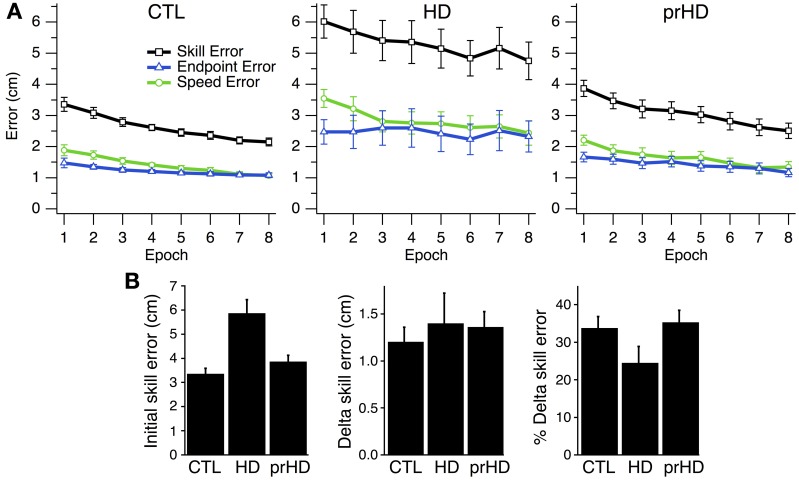
**Time course and group comparisons of skill learning. (A)** Group averages (mean ± SE) of skill error (squares, black trace), endpoint error (triangles, blue traces), and speed error (circles, green trace) vs. epoch (32 trials each) for the CTL (left), HD (middle) and prHD (right) groups. **(B)** Group averages (mean ± SE) of initial skill error, delta skill error and percent delta skill error.

At the group level, skill error and its components steadily decreased for CTL and HD groups (Figure 3A, left and middle). The HD group showed significantly larger initial skill error (mean ± SE 5.9 ± 1.9 cm) than the CTL group (3.3 ± 0.9; *p* < 0.001, *t*-test; Figure 3B, left). Thus, HD affects initial task performance, likely by impairing movement execution. Both the CTL and HD groups, on the other hand, showed evidence of improvement on the skill task with practice.

**Figure 4:**
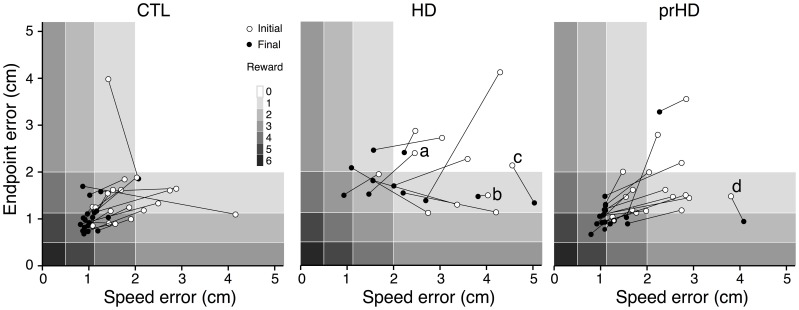
**Initial and final performance of individual subjects in task performance space**. The progression of skill error in performance space is shown as plots of initial (open circle) and final (closed circle) endpoint error vs. speed error for individual CTL (left), HD (middle) and prHD (right) subjects. Smiley face rewards (0–6) are indicated by varied shades of gray (light to dark).

Learning in individual subjects can be seen in a plot of endpoint error vs. speed error (*performance space*; Figure 4). The upper right region (low speed, low accuracy) corresponds to the least amount of skill, while the lower left region (high speed, high accuracy) reflects the most skilled performance. The remaining regions have some equivalence due to the task's speed-accuracy trade-off: it is similarly difficult to perform at low speeds with high accuracy (lower right region) and at high speed with low accuracy (upper left region). The task's reward structure (shaded rectangles in Figure 4) was designed to approximately reflect this trade-off: the greatest number of smiley faces (6) was shown for errors in the lower left region, while no smiley faces were shown for errors in the upper right region. Equivalent numbers of smiley faces could be obtained in corresponding upper left and lower right regions. A change in performance from the upper right toward the lower left region of performance space indicates a favorable change in the speed-accuracy trade-off, and thus an improvement of motor skill: movements become faster and more accurate, or faster with the same accuracy, or more accurate at the same speed.

CTL subjects' change in performance was mainly from the upper right to lower left regions of performance space (Figure 4, left), which indicates motor skill improvement. HD subjects' initial performance was worse than for CTL (open circles higher and further to the right in Figure 4, middle). Their final performance showed three patterns of change with practice. Some HD subjects showed evidence of motor skill learning with performance change toward the origin (Figure 4, middle, data point labeled a). Other HD subjects showed little change in performance (Figure 4, middle, point b), and some HD subjects showed a change that was mostly along the speed-accuracy trade-off (Figure 4, middle, point c).

### How can learning be compared between groups?

The difference in initial performance between CTL and HD groups is a potential confound in any analysis of learning (Soliveri et al., 1997). Group differences in learning could indicate a true disruption of the mechanisms that mediate skill improvement or a manifestation of movement execution deficits.

A few initial observations suggest that the learning process itself was disrupted by HD. First, in the case of an HD subject (Figure 2D) whose initial performance was better than that of a CTL subject (Figure 2C), the HD subject's error increased in early trials, whereas the CTL subject's error decreased. Second, this HD subject was able to occasionally make single movements that were very fast and accurate (dots in Figure 2D). This finding indicates that the ability to execute skilled movements was preserved, and that the impairment was in learning to perform these movements reliably. Third, the HD subject was able to reduce speed error or accuracy error separately, but unable to reduce them together (Figure 2D). This indicates that execution was sufficiently intact for the subject to control movement speed or movement accuracy individually, while respecting the speed-accuracy trade-off. The difficulty appeared to lie with reducing both errors together, i.e., with skill learning.

At the group level one could view the HD groups' learning curve as parallel to that of CTL (Figure 3A) and thus conclude that learning is normal for the HD group. Alternatively, one could note that the net reduction of skill error is a smaller fraction of initial error for the HD group that for CTL, because the HD group had larger initial error. These approaches to the learning results amount to comparing raw performance changes (delta values) or changes normalized to initial performance (percent changes). We examined both measures.

### Assessment of skill learning: means comparison

The first measure of learning that we considered, delta skill error, was not significantly different between HD (mean ± SE, 1.40 cm ± 1.12) and CTL groups (1.20 cm ± 0.67; *p* = 0.51, *t*-test; Figure 3B, middle). A second measure of learning, percent delta skill error, on the other hand, showed a trend toward reduced learning in the HD group (24% ± 15) compared to CTL (34% ± 13; *p* = 0.08, *t*-test; Figure 3B, right). While the percent change difference did not reach significance, it appears more sizable than the raw change. It is not clear which of these measures better captures learning. The difference between them may be partly understood by examining Figure 3A. The skill error traces for the CTL and HD groups are roughly parallel, which suggests that the raw delta change in error is not very different between groups. However, when the percent change is calculated, the large difference in initial performance yields a sizable difference in percent change between groups. Delta error accounts for initial performance differences by subtracting initial from final performance. Percent delta error, on the other hand, discounts initial differences multiplicatively. It would be desirable to establish some basis for choosing how to handle initial performance differences between groups.

### Assessment of skill learning: global regression

In order to address the difficulty in quantifying learning when initial performance differs, we employed a model-based analysis that exploited individual variation in initial performance to guide our method for discounting this variation across groups, as detailed in *Methods*. We considered linear and exponential processes as potentially underlying learning, as these processes explicitly embody the assumptions behind delta and percent delta measures. We first asked whether normal learning is better described by linear or exponential decay of error. We tested this by comparing linear and exponential models obtained through global regression of CTL subjects' learning curves, as detailed in *Methods*.

Quantifying learning in this way allowed us to determine which process and learning rate better described normal skill learning in CTL subjects. We then determined whether the HD group deviated from the normal learning process. Finally, because it was plausible that HD subjects might not show the same learning process as CTL subjects, but may still show evidence of improvement, we also separately determined which process and learning rates best characterized skill learning for the HD group separately.

**Figure 5:**
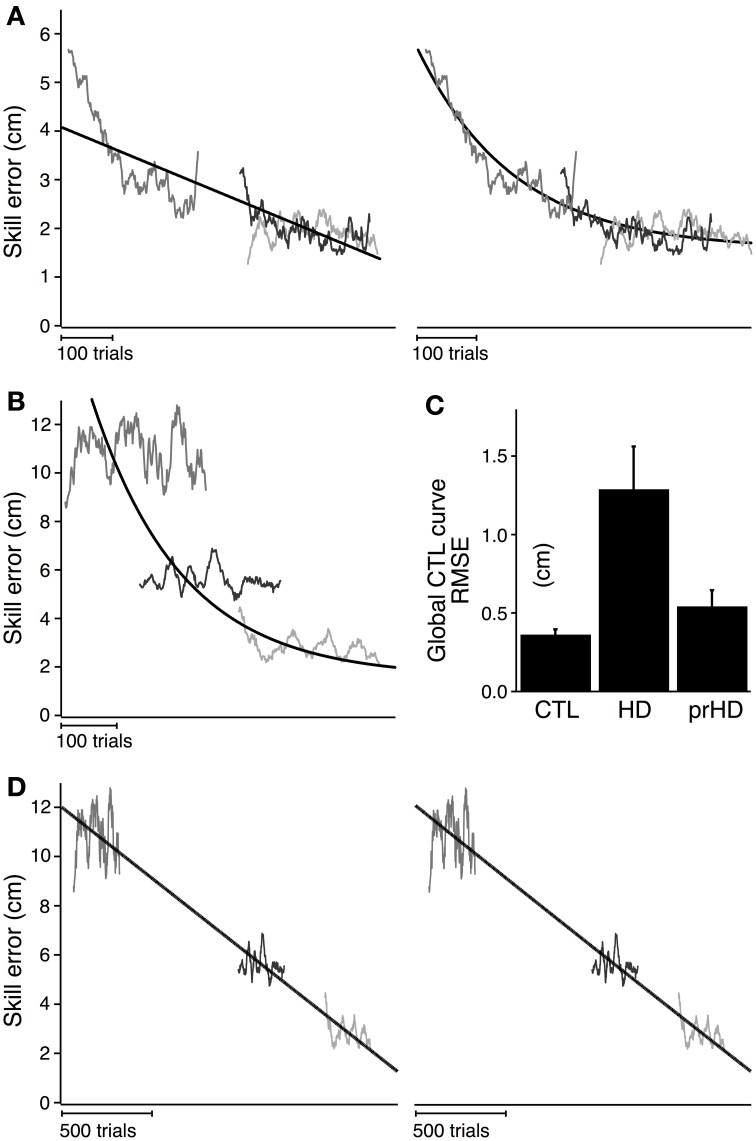
**Modeled time course of speed-accuracy learning. (A)** Black lines indicate the time course of skill error for the CTL group as modeled linearly (left; slope = −0.004; intercept = 3.19; AIC = 426) and exponentially (right; time constant = 162.4; amplitude = 2.08; AIC = 117). Smoothed skill errors of three sample CTL subjects (gray) are plotted such that RMSE is minimized. Their position along the *x*-axis is determined by the global fit process, which treats the learning curves for all subjects in a group as having the same learning rate but allows them to start at subject-specific initial error values. **(B)** Same exponential fit as **(A)** (black line), but with the smoothed skill errors of three sample HD subjects (gray). **(C)** Group averages (mean ± SE) of RMSE calculated with respect to the exponential learning curve for CTL. **(D)** Black lines indicate the time course of skill error for the HD group as modeled linearly (left; slope = −0.006 cm/trial; intercept = 5.88 cm; AIC = 4051) and exponentially (right; time constant = 62502 trials; amplitude = 364.1 cm; AIC = 4052). Smoothed skill errors of three sample HD subjects (gray) are plotted such that RMSE is minimized.

Figure 5A shows the global linear (left) and exponential (right) models for the CTL group. Goodness of fit for the CTL group was noticeably better for the exponential model (AIC = 117) than the linear model (AIC = 426). The time course of error for three single subjects illustrate the poor fit of the linear model (Figure 5A, left) and the better fit of the exponential model (Figure 5A, right).

We next determined whether skill learning for manifest HD subjects departed from the normal learning process by comparing all subjects in the HD group to the exponential learning curve for the CTL group. Learning curves for three representative HD subjects are superimposed on the exponential learning curve for the CTL group in Figure 5B. The starting position of each HD subjects' learning curve was determined by the global fit procedure (see *Methods*) to minimize RMSE. The process of fitting the α in Equation (2) to the data from the subjects, under the given values C and τ fitted to the CTL group, is equivalent to placing the individual HD subjects' skill-error data along the *x*-axis to minimize RMSE.

As Figure 5B shows, The HD subjects' learning curves deviate considerably from the normal learning curve. The average RMSE with respect to the global CTL learning curve was significantly higher for the HD group (1.3 ± 0.9 cm) than for the healthy CTL group (0.3 ± 0.1; *t*-test; *p* < 0.001). HD subjects' fit to the CTL curve was poor not only for subjects with large initial errors (for whom the normal curve was an extrapolation outside the range of CTL data), but also for those with initial errors in the same range as CTL.

It is not entirely surprising that the CTL group's data fits the normal curve better than the HD group's does, given that the normal curve was fit to CTL data. However, to the extent that the model reduces inter-subject variability due to differences in initial performance (as illustrated at least visually in Figure 5A, right panel), it suggests that the HD group's performance difference from CTL cannot be explained solely on the bases of initial performance impairments. This result also establishes a magnitude of deviation from normal learning, which may be compared to other groups (e.g., the prHD group as described below).

This difference in RMSE, however, does not describe how learning in HD is different—whether, for example, learning still follows an exponential process but with different parameters from CTL. To identify the nature of the learning disruption in HD, we conducted a separate global regression analysis for the HD subjects, independent of the performance of the CTL group. This analysis determined the learning rates and asymptotic behavior that were unique to the entire HD group, while allowing inter-subject variations in initial performance within the group. The results of the global linear and exponential models for the HD group (black line) are shown in Figure 5D. The time constant obtained in the exponential fit was so long that the fit curve does not look different from a line (Figure 5D, right). Skill errors were not well described by either the linear or exponential models: initial performance tended to be better than predicted by either model, while performance following practice tended to be worse than predicted (Figure 5D). The AIC was very large compared to the results obtained for CTL for both linear (AIC = 4051 for HD vs. 426 for CTL) and exponential (AIC = 4052 for HD vs. 117 for CTL) models.

These results demonstrate that neither the linear nor exponential models adequately explained the learning process for HD subjects, even with learning rates and asymptotes that were specific to that group. The results are further evidence that motor skill learning was disrupted in the HD group, beyond what might be explained by initial performance impairment.

### Progression of speed-accuracy performance during learning

**Figure 6:**
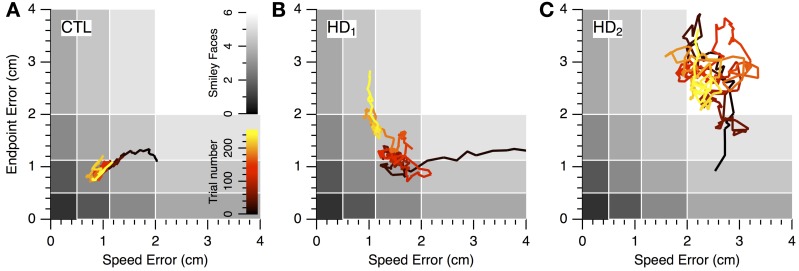
**Progression of speed-accuracy performance during practice**. The progression of skill error in performance space is shown by plots of endpoint error vs. speed error (smoothed with 16-trial window moving average). Trace color indicates trial order (early to late, black to yellow). Gray shading indicates number of smiley face rewards (0–6, light to dark). **(A)** Single CTL subject. **(B,C)** Single HD subjects.

The nature of the learning impairment can be visualized in the progression of individual subjects' endpoint and speed errors in performance space (Figure 6). The CTL subject in Figure 6A started at low speed and high accuracy (bottom right region of performance space) and progressed toward higher speeds with preserved accuracy. This progression follows a direction of improvement in both speed and accuracy that is parallel to a positive-slope diagonal in performance space. We refer to this direction (top right to bottom left) as a *skill-gradient* direction (solid arrow in Figure 6A).

An HD subject started with lower speed and lower accuracy (Figure 6B) but was able to improve into the same region of performance space as the CTL subject (red portions of traces in Figures 6A,B). However, with further practice the CTL subject improved its speed-accuracy further, while the HD subject's performance moved along the speed-accuracy trade-off, i.e., by increasing speed at the expense of accuracy or vice versa (yellow portion of traces in Figures 6A,B). This HD subject's performance trace followed, in the late stages of learning (yellow portion of trace in Figure 6B), a path that was tightly aligned with a negative-slope diagonal of performance space. We refer to this direction (top left to bottom right in performance space) as a *skill-contour* direction, because it likely indicates a contour line of performance limit imposed by the speed-accuracy trade-off. This behavior is consistent with exploratory attempts to improve performance by moving with different combinations of speed and accuracy that are all at the subject's performance limit, possibly in a search for ways to “break through” his/her own performance limit.

By contrast, another HD subject's performance (Figure 6C) unfolded into regions of lower accuracy regardless of changes in speed, and in varying directions rather than along a contour line of maximum performance. This pattern suggests a more severe disruption of learning because the subject was unable to explore performance space along his/her own performance limits. It also illustrates the separate nature of learning and execution deficits. This subject's initial performance is actually better than the CTL subject's and the other HD subject's, and yet learning progresses toward regions of worse performance.

### Relationship between changes in speed and accuracy

The core difficulty imposed by the motor learning task was the trade-off between speed and accuracy. Improving one of these variables at the expense of the other constitutes motor execution at the limit of one's performance, and does not indicate skill learning. The ability to concomitantly increase both speed and accuracy, and thus favorably change the speed-accuracy trade-off, was what we considered a manifestation of motor skill learning. Speed-accuracy learning should be manifested as a positive correlation between changes in speed and changes in accuracy across trials. Conversely, a negative correlation would indicate that movement execution is limited by the speed-accuracy trade-off, and that speed-accuracy learning is not occurring. Therefore, we examined the structure of speed-accuracy learning by testing for correlation between changes in speed error and changes in endpoint error from one trial to the next (as detailed in *Methods*).

**Figure 7:**
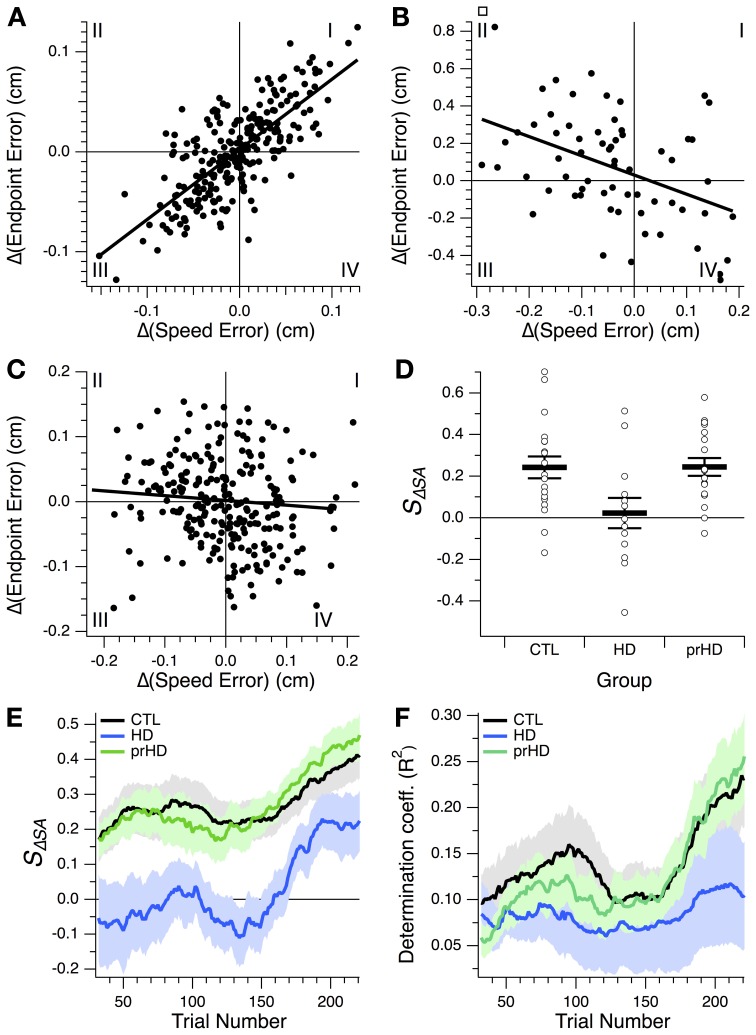
**Relationship between changes in speed and accuracy across trials. (A–C)** Trial-by-trial changes in endpoint error vs. changes in speed error for a CTL subject **(A)** and two HD subjects **(B,C)**. Linear regression lines are also shown. All 256 trials from the learning session are shown in **(A,C)**; the first 64 trials are shown in **(B)**. Roman numerals indicate standard numbering of quadrants in *x-y* plots. **(D)** Speed-accuracy-change slope, *S*_Δ*SA*_, for the three subject groups studied. This slope is the slope of the linear correlation between endpoint error changes and speed error changes, as illustrated in **(A–C)**. Horizontal bars indicate group mean ± SE. Open circles indicate individual subjects' slopes. **(E,F)** Progression of speed-accuracy-change slope, *S*_Δ*SA*_, and coefficient of determination, *R*^2^, for the linear correlation between trial-by-trial endpoint error change, Δ*(Endpoint Error)*, and speed error change, Δ*(Speed Error)*. These correlations were calculated, for each trial, based on a 64-trial window around that trial, which yielded values for trials 32–224 of the 256-trial learning session. Traces indicate group means (black, CTL; blue, HD; green, prHD). Shading denotes standard error.

A positive correlation is illustrated for a CTL subject in Figure 7A. This correlation, created by a predominance of points in quadrants I and III, corresponds to progression of learning along a skill-gradient direction in performance space (Figures 4, 6). Quadrant III contains reductions in both speed and accuracy errors, i.e., speed-accuracy improvements. Quadrant I contains increases in both errors, i.e., concomitant speed-accuracy decrements. A negative correlation is shown in Figure 7B for an HD subject (for illustration purposes, only the first 64 trials are shown). This plot is dominated by speed increases (reductions in speed error) that resulted in decreased accuracy (increased endpoint error; quadrant II). Similarly, improved accuracy occurs most commonly when speed decreases (quadrant IV). This pattern corresponds to changes in performance along the skill-contour direction in performance space (Figure 6B, yellow portion of trace). A third pattern, commonly observed among HD subjects, consisted of a more uniform distribution of points across all quadrants, but with smaller values in quadrant III than in the other quadrants (Figure 7C), indicating smaller speed-accuracy improvements (quadrant III) than speed-accuracy (quadrant I) decrements and trade-offs between speed and accuracy (II, IV). This pattern gave rise to small negative or positive correlation slopes.

We refer to the slope of the regression between endpoint error changes and speed error changes as the speed-accuracy-change slope, *S*_Δ*SA*_. This slope reflects the change in accuracy that accompanies a change in speed. At the group level this slope was on average positive for the CTL group and significantly greater than for the HD group (*p* = 0.017, 2-sample *t*-test; Figure 7D), which provides more detailed support for the finding that the HD group had impaired speed-accuracy learning.

We also examined the progression of the speed-accuracy change correlation over the course of learning by calculating the slope and the proportion of the variance accounted for by the linear regression (coefficient of determination, *R*^2^) for a sliding 64-trial window (Figures 7E,F). The slope increased in the second half of the learning session for both groups (Figure 7E), but, unlike for the CTL group, this change was not accompanied by an appreciable increase in *R*^2^. These results suggest that, over the course of learning, CTL subjects improved speed and accuracy in an increasingly coupled manner, more so than HD subjects did.

The presence of any data points in quadrant I is notable. These represent correlated degradations of both speed and accuracy from one trial to the next. If performance at every step were always dictated by the speed-accuracy trade-off, then one might expect data points only in quadrants II and IV (trade-off between speed and accuracy) and in quadrant III (coupled improvements in both variables). The presence of data points in quadrant I indicates that motor execution in a speed-accuracy learning task includes performance that is not at the speed-accuracy limit. This may be the result of normal movement variability or may reflect exploration of performance space in directions that are away from the current limit. We can only speculate as to whether this exploration could in turn reflect attempts to remodel movement execution in an effort to break through the current speed-accuracy barrier.

### Performance and skill learning of premanifest HD group

Overall, the prHD group's initial performance and skill learning were generally similar to those of CTL subjects. Their skill error values initially and throughout the course of learning were intermediate between those of CTL and HD groups (Figure 2A, right). At the individual subject level, several prHD subjects had poorer initial performance than that of CTL subjects (Figure 4, right). Their net performance change, however, was an increase in both speed and accuracy for all but one prHD subject (Figure 4, right, subject d). Thus, some amount of skill learning occurred for nearly all prHD subjects.

Comparisons of prHD to CTL for initial performance (*t*-test; *p* = 0.55), delta skill error (*p* = 0.55), and delta percent skill error (*p* = 0.74), did not identify statistically significant differences (Figure 3B). As we did for the HD group, in order to assess prHD subjects' entire time course of learning relative to the normal learning process, we compared skill errors of the prHD subjects to the global CTL exponential curve (obtained through the global regression approach). The mean RMSE for the prHD group was not significantly different from that of the CTL group (*p* = 0.31; Figure 5C).

With regard to correlations between changes in speed and accuracy across trials, the prHD group had a positive speed-accuracy slope that was indistinguishable from that of the CTL group (*t*-test; *p* = 0.98; Figure 7D). The progression of this slope and its related coefficient of determination through the learning session was similar to that of the CTL group (Figures 7E,F).

These results suggest the possibility of slight deficits in motor execution and skill learning that might occur prior to the diagnosis of HD, but that were not detectable at the group level (see also the next section).

### Correlation with disease severity and time before diagnosis

**Figure 8:**
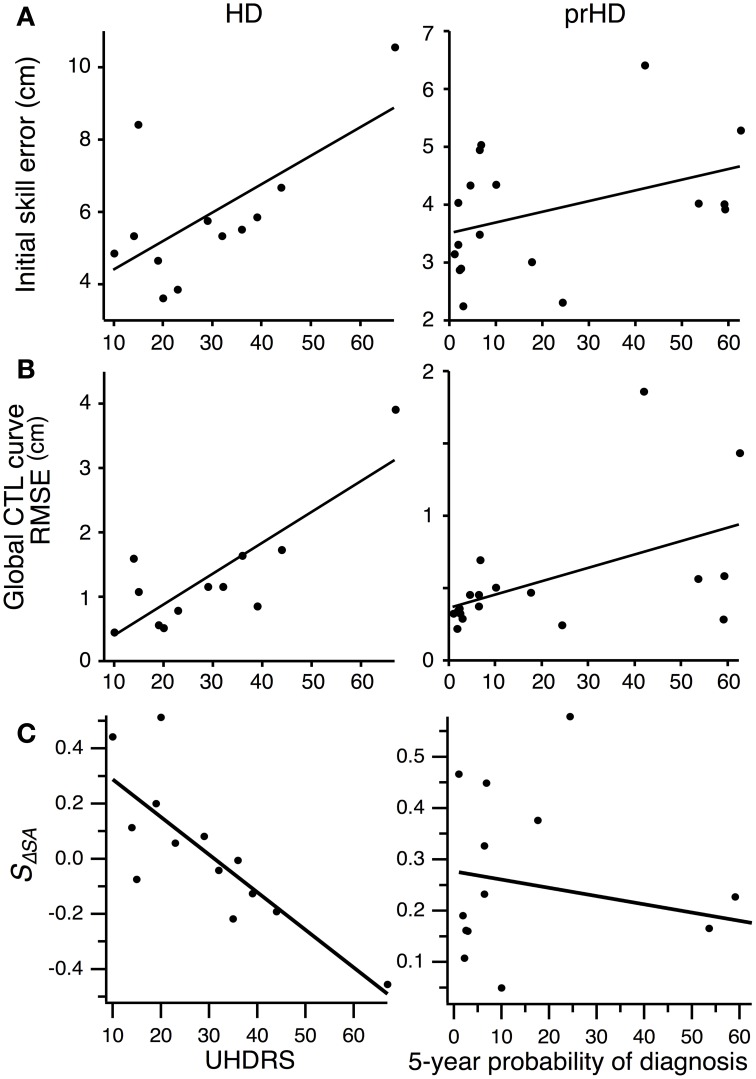
**Correlation between motor performance and disease severity for HD subjects, and between motor performance and proximity to symptom onset for prHD subjects**. Each point represents individual subject averages. Lines indicate linear regressions. **(A)** Relationship between initial skill error and UHDRS for HD subjects (left), and between initial skill error and 5-year probability of diagnosis for prHD subjects (right). **(B)** Relationship between the RMSE calculated with respect to the global exponential CTL curve and UHDRS for HD subjects (left), and between RMSE and 5-year probability of diagnosis for prHD subjects (right). **(C)** Relationship between speed-accuracy-change slope (*S*_Δ*SA*_) and UHDRS score for HD subjects (left), and between this slope and 5-year probability of diagnosis for prHD subjects (right).

We asked whether impairments in initial performance might reflect overall disease severity in HD subjects by performing linear regression between initial skill errors and UHDRS scores. The result was unclear due to the inordinate contribution of an outlying data point (Figure 8A, left). The correlation was significant (*R*^2^ = 0.42; *p* < 0.01) if we included this data point, but not significant if we excluded it (*R*^2^ = 0.01; *p* = 0.74). The Spearman rank correlation, which is less sensitive to outliers, was not significant (Spearman's ρ = 0.42; *p* = 0.17; all data points included).

We obtained a similarly mixed result for the correlation between skill learning, assessed by RMSE from the normal CTL learning curve, and UHDRS score. The correlation was significant if the outlier was included (*R*^2^ = 0.68; *p* = 0.001; Figure 8B, left), but not if the most affected subject was excluded (*R*^2^ = 0.19; *p* = 0.18). The Spearman rank correlation was not significant (Spearman's ρ = 0.52; *p* = 0.08).

There was a strong negative correlation, however, between the speed-accuracy-change slope (*S*_Δ*SA*_) and the UHDRS score. This correlation was significant whether the most affected subject was included (*R*^2^ = 0.64; *p* = 0.001) or not (*R*^2^ = 0.50; *p* = 0.007). The speed-accuracy-change slope is a specific indicator of subjects' ability to improve their speed-accuracy trade-off, with negative values reflecting reduced ability to improve both speed and accuracy simultaneously (as illustrated in Figure 7B). We interpret these results to mean that motor skill learning is impaired by HD in proportion to the disease's overall severity.

For prHD subjects there was no significant relationship between initial performance and an estimate of how close subjects were to the time of disease onset (probability of diagnosis in 5 years) (*R*^2^ = 0.15; *p* = 0.11; Figure 8A, right). However, extent of skill learning showed a small but significant negative correlation with 5-year probability of diagnosis (*R*^2^ = 0.26; *p* < 0.05; Figure 8B, right): prHD subjects who were closer to the time of disease onset achieved less skill learning than those who were farther from disease onset. This finding was not, however, reflected in the prHD group's speed-accuracy-change slope, which did not have a significant correlation with 5-year probability of diagnosis (*R*^2^ = 0.04; *p* = 0.41; Figure 8C, right).

## Discussion

We approached the question of how disruption of frontostriatal neural circuits affects motor skill learning. Two important features of the present study were its emphasis on how to define and measure motor skill learning, and on how to disambiguate motor execution impairment from motor learning abnormalities. We focused on these aspects in order to address longstanding limitations in our knowledge of basal ganglia function due to, respectively, variation in definitions of motor skill learning (definition confound) and the confounding effect of movement impairment on motor learning (performance confound). We addressed the definition confound by studying a specific component of skilled movement execution, i.e., making fast and accurate reaching movements, and measured motor skill learning as performance improvement in a speed-accuracy task. This choice allowed us to focus of motor skill learning as acquisition of a new level of motor ability (moving faster without loss of accuracy, and moving more accurately at a given speed), separate from other types of motor learning such as adaptation and sequence learning. We addressed the performance confound by modeling the effect of initial performance on motor learning in control subjects, and using this model to factor out initial performance from the patients' learning measures. This approach allowed us to assess learning separately from initial performance deficits.

We found that HD disrupts both the execution of skilled reaching movements and the improvement of fast accurate movements through learning. The disruption in execution was reflected in HD subjects' initial performance, which was characterized by movements that were both slower and less accurate than normal. Learning abnormalities were evident as deviation from the normal learning curve, which was well described by an exponential decay of performance error. These findings support a role for the basal ganglia in both movement execution and motor skill learning.

### Type of motor learning studied

Previous studies of the effect of basal ganglia disorders on motor learning likely addressed different types of motor learning, including motor adaptation, motor sequence learning, and motor skill learning. Consideration of these differences may explain some of the variation in these studies' conclusions.

Motor adaptation (Shadmehr et al., 2010) is a gradual change in the relationship between motor commands and sensory signals in response to an external perturbation, such as an external force or a change in the visuomotor map (Mazzoni and Krakauer, 2006; Tseng et al., 2007). The goal of adaptation is to return performance to baseline by counteracting the perturbation. Studies that have reported normal motor learning in HD and PD subjects mostly employed adaptation tasks. These include adaptation to laterally displacing prisms (Fernandez-Ruiz et al., 2003) (HD, PD subjects), horizontally and vertically inverted mappings between a visual display and movements on a digitizing tablet (Boulet et al., 2005) (HD), laterally displacing forces (Smith and Shadmehr, 2005) (HD, prHD), and visuomotor rotations (Mazzoni and Wexler, 2009) (prHD) (Marinelli et al., 2009; Bedard and Sanes, 2011) (PD). The latter two studies reported abnormalities of savings (rate of learning on a second exposure to a perturbation), but savings likely reflects a separate process from adaptation (Huang et al., 2011); adaptation on first exposure to the perturbation was normal. One study did report abnormal adaptation to visuomotor rotation in PD subjects (Contreras-Vidal and Buch, 2003). However, this study tested the effect of imposing a large rotation (90°), far larger than the rotations employed in most studies of visuomotor adaptation. Learning large rotations likely requires additional motor learning processes besides adaptation (Abeele and Bock, 2001). Indeed, a follow-up study confirmed that adaptation to small rotations is normal in PD (Venkatakrishnan et al., 2011).

In motor sequence learning, the elements of a sequence of movements are learned through practice (Nissen and Bullemer, 1987; Doyon et al., 2009). These tasks typically require an improvement in recall of what movements to select, and do not specifically reward improvements of the quality of individual movements. Motor sequence learning is generally abnormal in HD and PD (Knopman and Nissen, 1991; Ferraro et al., 1993; Pascual-Leone et al., 1993; Jackson et al., 1995; Ghilardi et al., 2008).

Several reports of abnormal motor learning in HD and PD subjects used tasks that did not require adaptation or sequence learning. We argue that the tasks used in these studies tested a similar component of motor skill learning to what we measured in the present study, namely, improvement in moving rapidly and accurately. The “rotor pursuit” task, for example (Heindel et al., 1988, 1989; Soliveri et al., 1992, 1997; Willingham and Koroshetz, 1993; Gabrieli et al., 1997) required subjects to hold a stylus over a small disc on a turntable, keeping the stylus in contact with the disc as much as possible. This task imposes a speed-accuracy trade-off by requiring subjects to maintain accuracy as the rotor spun at higher speeds. Deficits in learning this task have been identified in HD and PD subjects (Heindel et al., 1988, 1989; Gabrieli et al., 1997). One study reported normal learning for HD subjects in a joystick-guided version of this task (Willingham et al., 1996), but whether learning was truly normal may justifiably be questioned because the HD subjects' learning curve was appreciably different from that of controls. Another task used was a dual task, which consisted of fastening buttons on a cardigan while tapping one foot (Soliveri et al., 1992). While difficulty seems to be introduced by the dual nature of the task, it is revealing that the effect of the dual-task condition on performance was that each task was performed accurately but more slowly. This slowing suggests that a speed-accuracy trade-off existed in this task, and that PD subjects showed a more limited capacity to improve their speed-accuracy performance. Yet another task required subjects to track the movements of a screen target by controlling the velocity of a screen cursor through elbow flexion-extension (Soliveri et al., 1997). Although this task required learning a visuomotor transformation, a major component of difficulty was again imposed by a speed-accuracy trade-off: tracking the screen target was much easier when the target moved slowly than when it moved fast.

The impairment of speed-accuracy learning we found in HD patients is consistent with these previous results. We believe this commonality is due to the fact that tasks like the rotor pursuit used in these studies were in effect speed-accuracy tasks. These results differ from those of studies that tested motor adaptation. Unlike adaptation, speed-accuracy learning is not driven by a perturbation. Speed-accuracy learning is dominated by a reduction in movement variability, and is accompanied by changes in movement kinematics, such as increased trajectory smoothness, that indicate improved movement quality (Shmuelof et al., 2012). At the information-processing or computational level (Marr, 1982), reducing systematic error through adaptation and reducing variable error (improving movement reliability) through speed-accuracy training are potentially distinct operations, and it is thus not surprising that the basal ganglia contribute to them differently.

The idea that different forms of motor learning involve distinct processes has been previously suggested (Willingham et al., 1996; Gabrieli et al., 1997; Shmuelof and Krakauer, 2011). Gabrieli (Gabrieli et al., 1997) directly compared rotor pursuit learning, which was impaired in HD subjects, to mirror writing, which was normal. We suggest that mirror writing is a task that mainly requires adaptation, because learning is driven by a steady external perturbation of a sensorimotor mapping. If we thus account for differences in types of motor learning studied, our results support the idea that HD disrupts a specific type of motor learning, namely, the component of motor skill learning that is embodied by an improvement in speed-accuracy trade-off performance. The basal ganglia are thus an important neural substrate for some forms of motor learning, but not for others.

### Fast accurate movements and motor skill

It is not obvious how to define, in some general sense, the type of motor learning tested in these studies and in the present one. We have used the terms “speed-accuracy performance” and “speed-accuracy learning” as working labels in our study. It would be desirable to map these labels onto candidate psychological processes and neural computations that support them. The core of the improvement in speed-accuracy learning is to move faster and more accurately, i.e., to improve performance against the speed-accuracy trade-off imposed by the task and the neuromotor and biomechanical limitations of movement execution. Performance is limited by two conflicting movement variables, speed and accuracy, and what is learned is not just the ability to move fast or accurately but the ability to move with a more favorable trade-off between these variables. The speed-accuracy trade-off is such a prominent and widespread limitation to motor performance that speed-accuracy improvement has been considered by some as a defining manifestation of motor skill learning (Sanes et al., 1990; Reis et al., 2009; Shmuelof et al., 2012).

The term “motor skill” has, however, also been used more generally to refer to an overall motor ability that encompasses multiple components. The International Classification of Function (ICF), for example, refers to skill as performing “integrated sets of actions so as to follow rules, and to sequence and coordinate one's movements” (World Health Organization, 2001). Such a general-level description reminds us of the complex and general nature of skills. However, we believe that the disparate components of motor skill converge to produce movements with specific qualities. These are captured by definitions of motor skill suggested by multiple previous authors, which may be synthesized as the ability to reliably produce a particular movement with minimal variability and maximal accuracy, as well as with minimal time and maximal energetic efficiency (Guthrie, 1952; Welford, 1968; Willingham, 1998; Schmidt and Lee, 2005). The elements of minimal variability and maximal accuracy are well instantiated in motor behavior with a speed-accuracy trade-off, and are easily recognized as the requirements of many competitive sports. Therefore, we consider the ability to move fast and accurately as a core element of many types of skilled movements, while recognizing that motor skill may be reflected in other movement features too.

It may be useful, in this regard, to consider the evolution that term “adaptation” has undergone in the motor learning literature. Behavioral adaptation may be conceived in the general sense of any change in behavior in response to change in (external or internal) conditions, and in this sense may encompass instrumental learning, sensorimotor remapping, and reinforcement learning. However, the term “motor adaptation” has come to refer to cerebellum-dependent gradual adjustment of an internal model based on prediction error (Mazzoni and Krakauer, 2006; Tseng et al., 2007; Shadmehr et al., 2010). We would advocate for a similar transformation of the term “motor skill learning,” from referring to a general behavioral process that covers many different abilities, to embodying a specific motor process, such as speed-accuracy learning, that will map onto identifiable neural circuitry. An alternative would be to maintain a general meaning for “motor skill” and introduce a new term to refer to the ability to move fast and accurately. We previously suggested “motor acuity” for this purpose (Shmuelof et al., 2012), in analogy with the use of perceptual acuity as a measure of perceptual skill.

### Impairments of motor skill learning vs. motor execution

Impairments of motor skill learning must be distinguished from deficits in motor execution (Soliveri et al., 1997), which may or may not affect motor learning. Indeed, HD disrupts the execution of reaching movements (Hefter et al., 1987; Thompson et al., 1988; Bradshaw et al., 1992; Georgiou et al., 1997; Quinn et al., 1997, 2001; Bonfiglioli et al., 1998; Smith et al., 2000; Verbessem et al., 2002), but does not disrupt some forms of motor learning (Fernandez-Ruiz et al., 2003; Boulet et al., 2005; Smith and Shadmehr, 2005; Mazzoni and Wexler, 2009). Although motor execution was clearly abnormal in HD subjects in the present study, our results suggest that the impairment in speed-accuracy learning was not simply a consequence of abnormal motor control. HD subjects maintained the ability to execute single highly skilled movements (single dots in Figure 2D), but were less able than CTL subjects to do so with increasing reliability. This dissociation between motor execution and skill learning deficits was borne out in our global regression analysis.

One approach to address the confound of motor execution impairments has been to make the task easier for patients, so as to make initial performance similar across groups (Heindel et al., 1988; Gabrieli et al., 1997). The problem with this approach is that while the outcome variable was matched (e.g., time on target in a rotor pursuit task), the movement itself was different (slower for HD subjects). Thus, groups were compared on effectively different motor tasks, without establishing how control subjects would have learned if the task had also been made easier for them too. Another approach, widespread across the learning literature, has been to remove differences in initial performance between groups through normalization. However, it is generally unclear whether normalization should be subtractive (subtracting initial from final performance) or fractional (dividing final by initial performance). Choosing one or the other approach implies specific assumptions about the relationship between initial performance and learning—assumptions that are rarely explicitly tested.

We therefore used a novel approach to manage the confound of baseline impairment. We exploited inter-individual variations in initial performance for the CTL group and determined that a decaying exponential was an appropriate model for the normal learning process. This approach allowed us to discount differences in HD patients' initial performance by comparing the patient's learning curve to the appropriate segment of the normal learning curve. HD subjects' learning curves considerably deviated from normal, even when shifted to match initial performance. Indeed, linear or exponential models could not account for HD subjects' learning even when fit to their data alone. Our results therefore argue that HD causes a motor skill-learning deficit that is separate from the impairment in initial performance.

Discounting the effect of initial performance differences does not entirely remove the confound of movement execution impairments. Such impairments could impede learning in ways other than simply pushing back the starting point of learning. Practice, after all, requires movement execution, and abnormal execution could provide the learning process with inadequate or incorrect information. These potential higher-order interactions between execution and learning were not addressed by our study. It is worth noting, though, that learning is not guaranteed to be disrupted by poor performance. In a recent study of speed-accuracy learning, subjects who practiced movements at higher speeds (and thus with more errors) experienced similar improvements in their speed-accuracy trade-off to subjects who practiced at lower speeds (and thus with fewer errors) (Shmuelof et al., 2012). Analogously, Smith et al. showed that HD disrupts the execution of arm trajectories by blunting the amplitude of online error corrections (Smith et al., 2000). This execution impairment, however, did not affect HD subjects' ability to improve performance on a trial-by-trial basis (Smith and Shadmehr, 2005). In other words, motor learning was normal in spite of execution impairment.

It is notable in this regard that even the most impaired HD subjects occasional produced highly skilled movements. In order to invoke an execution deficit to explain their learning difficulties, we would need to consider the difference between the ability to execute a single fast accurate movement occasionally vs. reliably. Such a distinction has been explored with regard to the neural basis of movement variability (Churchland et al., 2006). Indeed, we expect that improved understanding of execution variability will help to formulate new hypotheses regarding how skilled motor performance is acquired. At this point, though, how abnormal execution interacts with learning, other than by affecting initial performance, is a question of fundamental interest that remains largely unanswered.

### Possible contributions of chorea and cognitive deficits

It is interesting to consider whether chorea could be responsible for the movement execution or learning impairments we observed. Chorea could theoretically push the hand away from its intended path and thus cause path irregularities that would reduce end-position accuracy and possibly also affect learning. However, observation of the subjects while they performed the task, and visual inspection of their hands' velocity profiles, did not reveal clear instances of single sudden deviations.

It is important to note that the task in our study consisted of making very fast brief movements of the entire arm. These movements caused high accelerations and decelerations of the arm, and thus created inertial changes that would be unlikely to be affected by the relatively small amplitudes of the impulses typically associated with chorea, at least in our casual observation in the laboratory. Moreover, the task required a single rapid accurate movement, because the endpoint square was shown at the first velocity minimum. Submovements and fine adjustments of hand positions, which are more likely to be affected by chorea, could not contribute to improved performance and, indeed, were not observed. For these reasons, we do not believe that chorea appreciably contributed to the motor execution or learning deficits observed in the present study.

We cannot exclude the possibility that cognitive deficits, which are part of the symptoms caused by HD, might contribute to the motor learning impairment observed. However, it is unlikely that cognition plays much of a role in the task we used. Improvement in the speed-accuracy trade-off consists mainly of gradual refinement of control of low-level kinematic variables, such as timing and amplitude of hand acceleration (Shmuelof et al., 2012), that are likely outside cognitive control.

### Performance of premanifest HD subjects

The performance of prHD subjects provided an opportunity to assess whether subtle deficits in motor skill learning emerge prior to the time of clinical diagnosis. Previous studies have described abnormalities of movement execution (De Boo et al., 1997; Kirkwood et al., 1999, 2000; Smith et al., 2000; Farrow et al., 2006; Rao et al., 2008; Biglan et al., 2009; Tabrizi et al., 2009), dual-task motor control (Mazzoni and Wexler, 2009), and sequence learning (Ghilardi et al., 2008). At the group level, we found that prHD subjects performed similarly to CTL subjects in motor execution and skill learning.

It is crucial to note, however, that the prHD group was heterogeneous and included subjects that were nearer to and farther from the expected time of onset of HD symptoms. Of particular interest, therefore, is the presence of an inverse correlation between motor skill learning and the 5-year probability of diagnosis, and the absence of such a correlation for initial performance. These findings suggest a possible time course of motor deficits for HD in which motor skill learning is disrupted prior to motor execution. Tests of motor skill learning might thus provide an earlier means for detection of disease onset than current clinical and neuropsychological methods, which generally assess motor execution.

### Relationship to disease severity

We found evidence of a correlation between speed-accuracy learning and degree of clinical severity in HD subjects. Such a correlation supports the idea that skilled movements and clinical signs result from a common pathophysiology. It is important, however, to keep in mind the pitfalls that accompany any attempt to correlate kinematic data with clinical severity scales. The absence of such a correlation would not exclude a relationship between kinematic findings and the underlying disease process, because clinical scales are imperfect markers of the underlying disease process [see, for example, (Pillai et al., 2012)].

### Potential implications for therapy

Therapies for patients with HD remain limited to medications that reduce some symptoms, and physical therapy which can reduce the impact of motor symptoms on daily function. Until these types of therapies become more specifically targeted toward learning impairments, our findings offer little guidance in the choice of current therapies. However, our findings have potential relevance to future rehabilitation strategies, as these become increasingly informed by our understanding of motor learning. To the extent that they shed light on the functions of the neural circuits affected by HD, our results may also be important to consider in the future design of neural prostheses to replace these circuits.

### Implications for striatal function

We identified a specific type of motor learning deficit, in addition to impaired movement execution, in subjects with HD. These findings suggest a role for frontostriatal circuits in motor skill acquisition, in addition to their known contribution to sequence learning. Whether the crucial component of frontostriatal circuits is the striatum itself is a difficult question. There is ample evidence that the motor cortex plays an important role in motor skill learning (Nudo et al., 1996; Kleim et al., 1998; Luft and Buitrago, 2005; Metz et al., 2005; Whishaw et al., 2008; Hosp et al., 2011). Furthermore, HD is known to cause degeneration of cortical motor areas in addition to the striatum (Rosas et al., 2002). The striatum could contribute to motor skill learning through its dopamine-dependent modification of corticostriatal synapses (Wickens and Kotter, 1995; Surmeier et al., 2007), specifically through reinforcement of corticostriatal signals that lead to more reliable production of successful movements. Alternatively, corticostriatal signals could guide circuit changes within the motor cortex, such as expansion of cortical representations of particular movements (Nudo et al., 1996).

Deciphering the role of the striatum in motor skill learning will require better understanding of how motor performance improves in speed-accuracy learning. In the case of motor adaptation, we know that some modification of an internal model for movement planning must occur, with a central contribution by the cerebellum. By contrast, the computational and neural processes required to improve speed-accuracy performance and, more generally, skilled movement execution, remain mysterious. Parallel approaches to patient studies, such as computational techniques and neurophysiology, will hopefully converge with our results to shed further light on this question.

### Conflict of interest statement

The authors declare that the research was conducted in the absence of any commercial or financial relationships that could be construed as a potential conflict of interest.
